# Electrical Instruments

**Published:** 1873-03

**Authors:** 


ELECTRICAL INSTRUMENTS.—In the interest which has recently been aroused in regard to the therapeutic uses, of electricity the general inquiry has been, where shall I get a suitable instrument. The general desire has been to possess an instrument which was not so complicated as to be continually getting out of order and yet was capable of producing the desired effect.

The Galvano-Faradic Manufacturing Company of New York offer for sale Electro-Magnetic Machines, which for beauty of construction, and promptness of action are unsurpassed. These machines are provided with all the modern improvements, and are durable, light and portable. The currents produced by these machines are perfectly under the control of the operator and can be applied to the most delicate organizations without producing the disagreeable feeling which so often attends the use of electricity. We have been using one of these machines for some time and have been uniformly pleased with its action.

The Galvanic and Galvano-Caustic Batteries manufactured by this Company are models of excellent workmanship. We have had two or three occasions to use their Galvanic Battery, and were pleased at the perfect control which we had over the current, being enabled at will to make it stronger or weaker.

These machines are so constructed that it is not necessary to remove the fluid from the cells when transporting the instruments, it being perfectly confined within the cells. Several of these instruments are in use in this locality, and we have always heard them spoken of in the highest terms.

